# The burden of new onset atrial fibrillation in the intensive care unit (ICU). an observational, prospective, single-center study

**DOI:** 10.1186/2197-425X-3-S1-A212

**Published:** 2015-10-01

**Authors:** A Papathanasiou, E Roustanis, X Zikou, E Galiatsou, G Papathanakos, G Zilis, M Saranti, V Koulouras, A Karachaliou, G Nakos

**Affiliations:** University Hospital of Ioannina, Dept of ICU, Ioannina, Greece

## Intr

Cardiac arrhythmias constitute a great challenge in the ICU, since they aggravate a borderline cardiopulmonary function. Atrial fibrillation (AF) forms the commonest type of arrhythmias and an unappreciated chapter of the respective literature at the same time.

## Objectives

To estimate the prevalence of new onset AF in ICU patients.

## Methods

the study took place in a 13 level-3 bed ICU, of a university hospital in Northwestern Greece, during December 2013-September 2014. All admitted patients were scrutinized and their medical history, APACHE II score, admission and discharge diagnosis, AF prevalence along with arterial blood gases (ABGs) and vital signs during the attack, arrhythmia´s treatment outcome and any possible damaging factors responsible for its appearance were recorded.

## Results

A total of 192 patients were admitted during the study period (124 men, 68 women, mean age 59.2 and 59.8 years respectively). Total mortality was 27.6%, mean ICU staying was 15 days (1-77 days). Twelve patients had history of chronic AF (CAF) and 7 had history of paroxysmic AF (PAF). During hospitalization, 37 patients developed rapid ventricular response AF (RVAF, 7/12-58.33% with CAF, 4/7-57.14% with PAF and 18/173-10.4% with no history of arrhythmias). Main factors for RVAF, common in all three groups, were hypoxemia, sepsis and septic shock, severe electrolyte abnormalities and APACHE II score above 30. Amiodarone (300 mg in 30 minute infusion, followed by either 900 mg in 24-hour infusion or 300 mg tid) was the drug of choice in 32/37 patients, while in 5/37 patients (all with CAF and RVAF), iv b-blocker was initially administered. In 4/5 with CAF and RVAF, b-blocker was ineffective and amiodarone was additionally administered. In all patients with CAF and RVAF, amiodarone was effective for rate control. Amiodarone was also the drug of choice for rhythm restoration in all RVAF and history of PAF and those with no history of arrhythmias. Restoration of rhythm was successful in all of those patients. In all cases, RVAF improved quicker when the underlying condition was hypoxemia, rather than sepsis or septic shock, while electrolyte abnormalities rendered RVAF especially resistant to treatment, until those abnormalities were effectively addressed. Moreover, CAF patients were proven more difficult to respond to drugs than PAF ones. In most cases, initial 300 mg of amiodarone sufficed for RVAF control.

## Conclusions

RVAF is frequent in ICU patients, especially among those with prior history of cardiac disease. Proper treatment requires immediate control of the predisposing factor and drug regimen initiation, mainly amiodarone.

